# The genome sequence of the rose sawfly *Arge pagana* Linnaeus, 1758

**DOI:** 10.12688/wellcomeopenres.23289.1

**Published:** 2024-10-29

**Authors:** Liam M. Crowley, Andrew Green, University of Oxford and Wytham Woods Genome Acquisition Lab, Darwin Tree of Life Barcoding collective, Wellcome Sanger Institute Tree of Life Management, Samples and Laboratory team, Wellcome Sanger Institute Scientific Operations: Sequencing Operations, Wellcome Sanger Institute Tree of Life Core Informatics team, Tree of Life Core Informatics collective, Darwin Tree of Life Consortium

**Affiliations:** 1 University of Oxford, Oxford, England, UK; 2 Sawfly Recording Scheme, Bedford, England, UK

**Keywords:** Arge pagana, large rose sawfly, genome sequence, chromosomal, Hymenoptera

## Abstract

We present a genome assembly from a female specimen of *Arge pagana* (large rose sawfly; Arthropoda; Insecta; Hymenoptera; Argidae). The genome sequence has a total length of 204.70 megabases. Most of the assembly (94.4%) is scaffolded into 8 chromosomal pseudomolecules. The mitochondrial genome has also been assembled and is 26.8 kilobases in length.

## Species taxonomy

Eukaryota; Opisthokonta; Metazoa; Eumetazoa; Bilateria; Protostomia; Ecdysozoa; Panarthropoda; Arthropoda; Mandibulata; Pancrustacea; Hexapoda; Insecta; Dicondylia; Pterygota; Neoptera; Endopterygota; Hymenoptera; Tenthredinoidea; Argidae; *Arge*; *Arge pagana* Linnaeus, 1758 (NCBI:txid1384777).

## Background

The Argidae are the second largest family of sawflies globally. They are stocky, slow flying insects characterised by having the flagella segments of the antennae fused into a single filament. In males the flagellum either has a brush of setae on the underside or is shaped like a tuning fork. The genus *Arge* is distinguished from other genera within the family by having an enclosed apical cell in both the fore and hindwing and the hind tibia has a pre-apical spine. In the males, the antennae are simple. There are over 250 species of *Arge* globally and they are the dominant genus in tropical regions of Africa. There are 13 species in Britain and seven in Ireland ( Green, 2021; Liston *et al.*, 2014).

*Arge pagana* has two recognised subspecies: *pagana pagana* (Panzer, 1797) and *pagana stephensii* (Leach, 1817). Whilst the subspecies *pagana* is widespread across the Palaearctic, *stephensii* is endemic to Britain. In specimens throughout its range *pagana pagana* has the legs, mesonotum and mesopleura consistently black. However, specimens of *pagana stephensii* exhibit varying amounts of yellow on the legs, mesonotum and mesopleura ( Benson, 1950). This female specimen from Wytham Woods, England matches the description of *Arge pagana stephensii* using the characteristics in Benson’s key ( Benson, 1952), and the publication of the complete gene sequence will help our understanding of the phylogeny of this group.

*A. pagana* imagines are 7 to 9 mm and will often visit umbellifer flowers for nectar or pollen. As such they can be considered as casual pollinators. The larvae feed gregariously on roses including garden varieties. They are not considered to be a pest of agricultural or horticultural significance though they can be a nuisance for gardeners. The species is multivoltine with adults on the wing from May to September.

In BOLD ( BOLD, 2023), COI barcoding produces two clusters: BIN AAN1707 and BIN ABV4024. Based on the morphological distinctions, the subspecies *pagana* appears to be spread across both clusters and a single *stephensii* specimen from Britain is in BIN ABV4024.

## Genome sequence report

**Figure 1.**
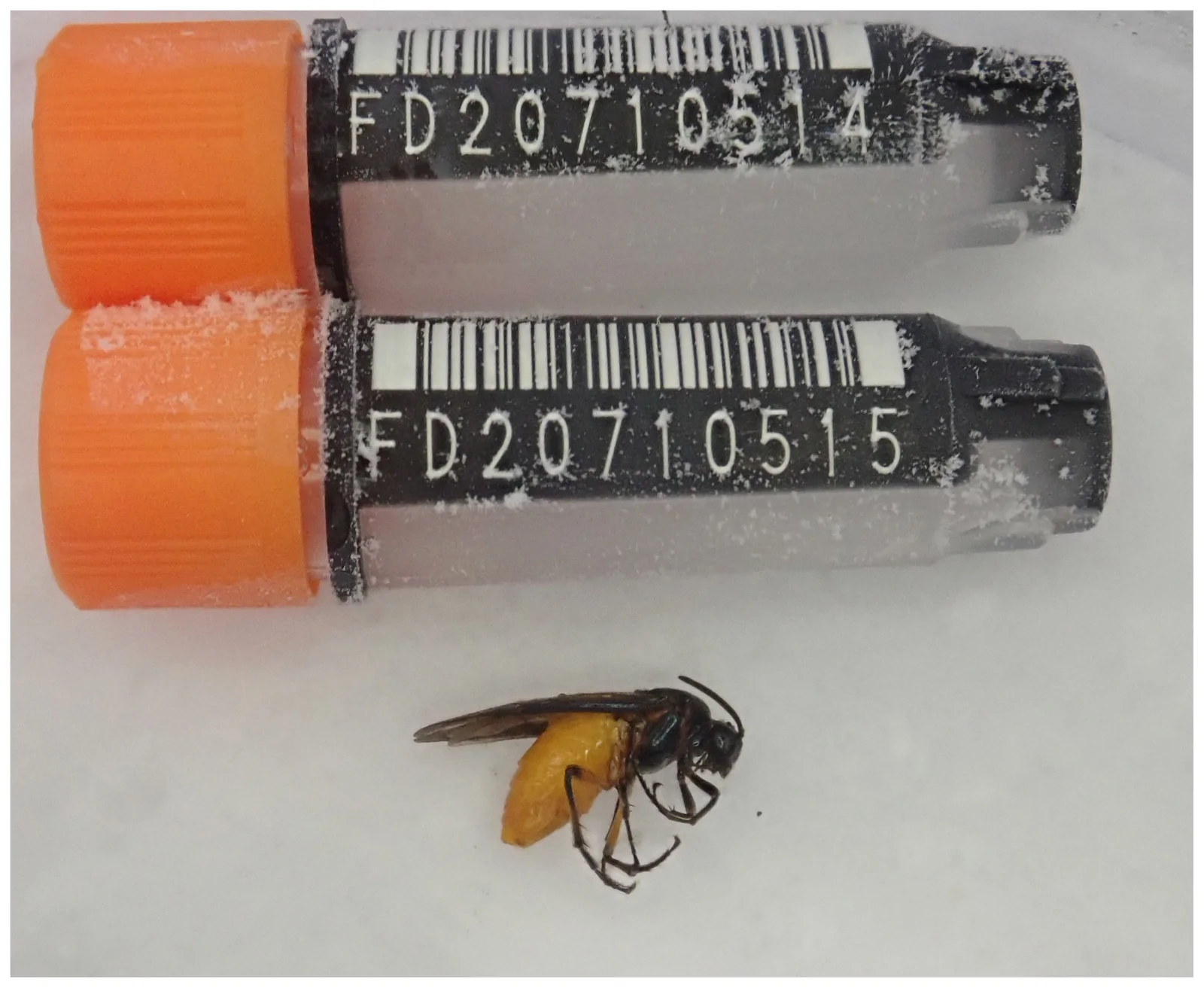
Photograph of the *Arge pagana* (iyArgPaga1) specimen used for genome sequencing.

The genome of *Arge pagana* ( Figure 1) was sequenced using Pacific Biosciences single-molecule HiFi long reads, generating a total of 20.41 Gb (gigabases) from 2.32 million reads, providing an estimated 104-fold coverage. Primary assembly contigs were scaffolded with chromosome conformation Hi-C data, which produced 135.35 Gb from 896.35 million reads, yielding an approximate coverage of 661-fold. Specimen and sequencing details are summarised in Table 1.

**Table 1.** Specimen and sequencing data for *Arge pagana*.

Project information			
**Study title**	Arge pagana (large rose sawfly)		
**Umbrella BioProject**	PRJEB66087		
**Species**	*Arge pagana*		
**BioSample**	SAMEA10978972		
**NCBI taxonomy ID**	1384777		
Specimen information			
**Technology**	**ToLID**	**BioSample accession**	**Organism part**
**PacBio long read sequencing**	iyArgPaga1	SAMEA10979332	Abdomen
**Hi-C sequencing**	iyArgPaga1	SAMEA10979331	Head and thorax
Sequencing information			
**Platform**	**Run accession**	**Read count**	**Base count (Gb)**
**Hi-C Illumina NovaSeq 6000**	ERR12071285	8.96e+08	135.35
**PacBio Sequel IIe**	ERR12055607	2.32e+06	20.41

Assembly errors were corrected by manual curation, including 179 missing joins or mis-joins and four haplotypic duplications. This reduced the scaffold number by 31.69% and increased the scaffold N50 by 113.27%. The final assembly has a total length of 204.70 Mb in 221 sequence scaffolds, with 545 gaps, and a scaffold N50 of 26.5 Mb ( Table 2).

**Table 2.** Genome assembly data for *Arge pagana*, iyArgPaga1.1.

Genome assembly		
Assembly name	iyArgPaga1.1	
Assembly accession	GCA_963969455.1	
*Accession of alternate haplotype*	*GCA_963969465.1*	
Span (Mb)	204.70	
Number of contigs	767	
Number of scaffolds	221	
Longest scaffold (Mb)	31.66	
Assembly metrics *		*Benchmark*
Contig N50 length (Mb)	0.9	*≥ 1 Mb*
Scaffold N50 length (Mb)	26.5	*> chromosome N50*
Consensus quality (QV)	55.9	*≥ 40*
*k*-mer completeness	99.99%	*≥ 95%*
BUSCO v5.4.3 lineage: hymenoptera_odb10	C:92.8%[S:92.5%,D:0.3%], F:1.8%,M:5.4%,n:5,991	*S > 90%* *D < 5%*
Percentage of assembly mapped to chromosomes	94.4%	*≥ 90%*
Sex chromosomes	None	*localised homologous pairs*
Organelles	Mitochondrial genome: 26.8 kb	*complete single alleles*

* Assembly metric benchmarks are adapted from Rhie *et al.* (2021) and the Earth BioGenome Project Report on Assembly Standards September 2024.

**Figure 2.**
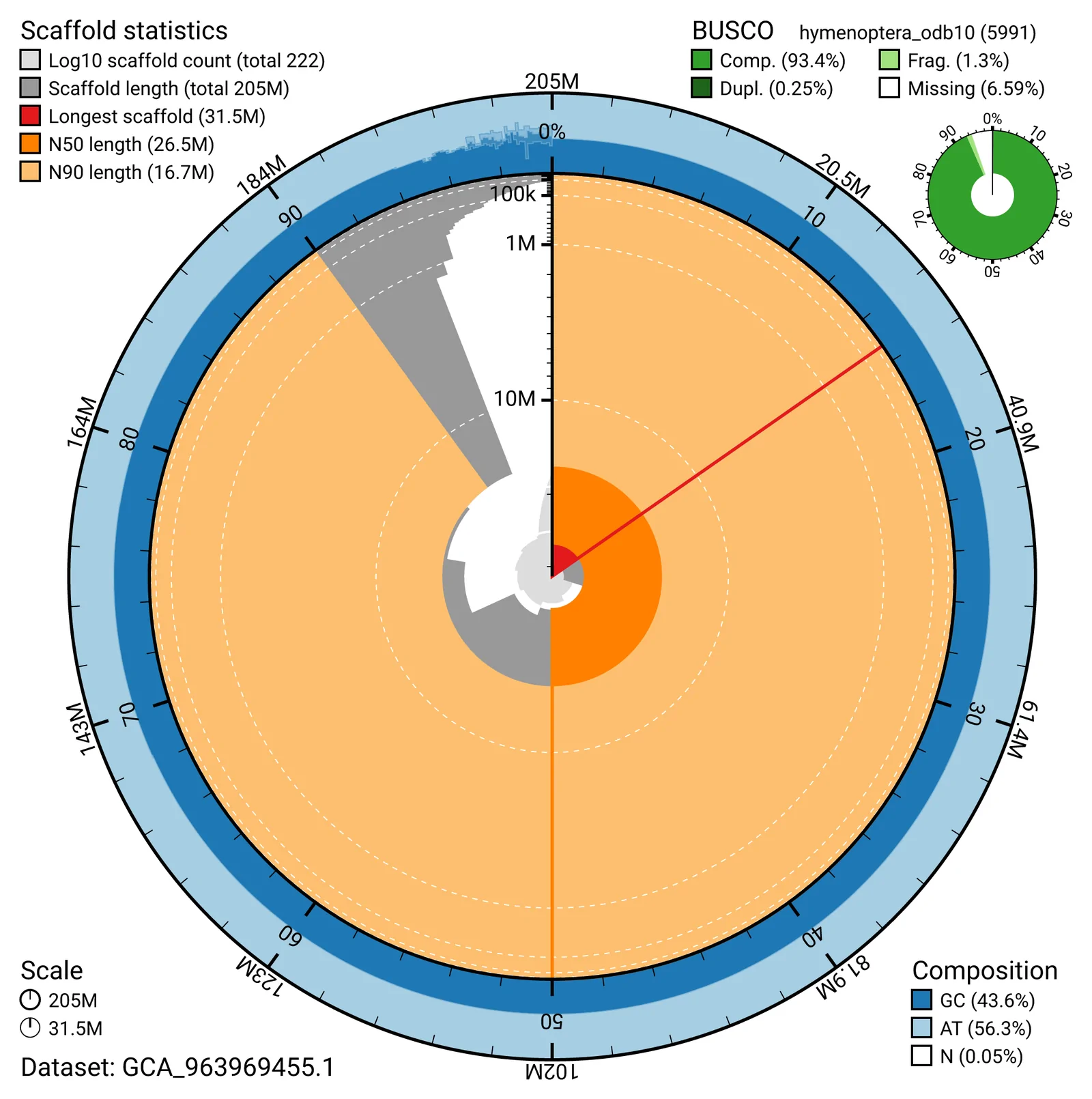
Genome assembly of *Arge pagana*, iyArgPaga1.1: metrics. The BlobToolKit snail plot shows N50 metrics and BUSCO gene completeness. The main plot is divided into 1,000 size-ordered bins around the circumference with each bin representing 0.1% of the 204,694,620 bp assembly. The distribution of scaffold lengths is shown in dark grey with the plot radius scaled to the longest scaffold present in the assembly (31,496,704 bp, shown in red). Orange and pale-orange arcs show the N50 and N90 scaffold lengths (26,513,337 and 16,683,171 bp), respectively. The pale grey spiral shows the cumulative scaffold count on a log scale with white scale lines showing successive orders of magnitude. The blue and pale-blue area around the outside of the plot shows the distribution of GC, AT and N percentages in the same bins as the inner plot. A summary of complete, fragmented, duplicated and missing BUSCO genes in the hymenoptera_odb10 set is shown in the top right. An interactive version of this figure is available at https://blobtoolkit.genomehubs.org/view/GCA_963969455.1/dataset/GCA_963969455.1/snail.

**Figure 3.**
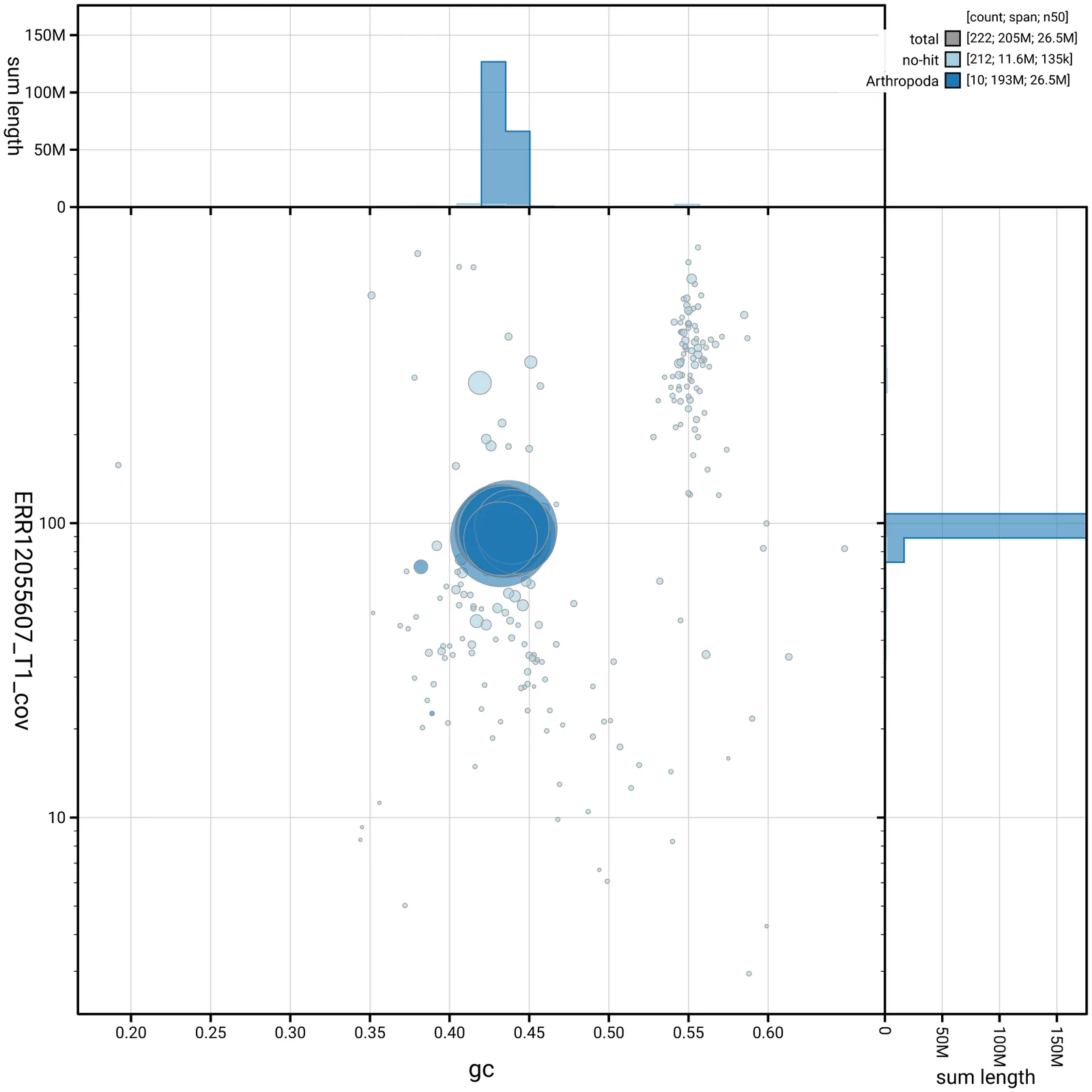
Genome assembly of *Arge pagana*, iyArgPaga1.1: BlobToolKit GC-coverage plot showing sequence coverage (vertical axis) and GC content (horizontal axis). The circles represent scaffolds, with the size proportional to scaffold length and the colour representing phylum membership. The histograms along the axes display the total length of sequences distributed across different levels of coverage and GC content. An interactive version of this figure is available at https://blobtoolkit.genomehubs.org/view/GCA_963969455.1/dataset/GCA_963969455.1/blob.

**Figure 4.**
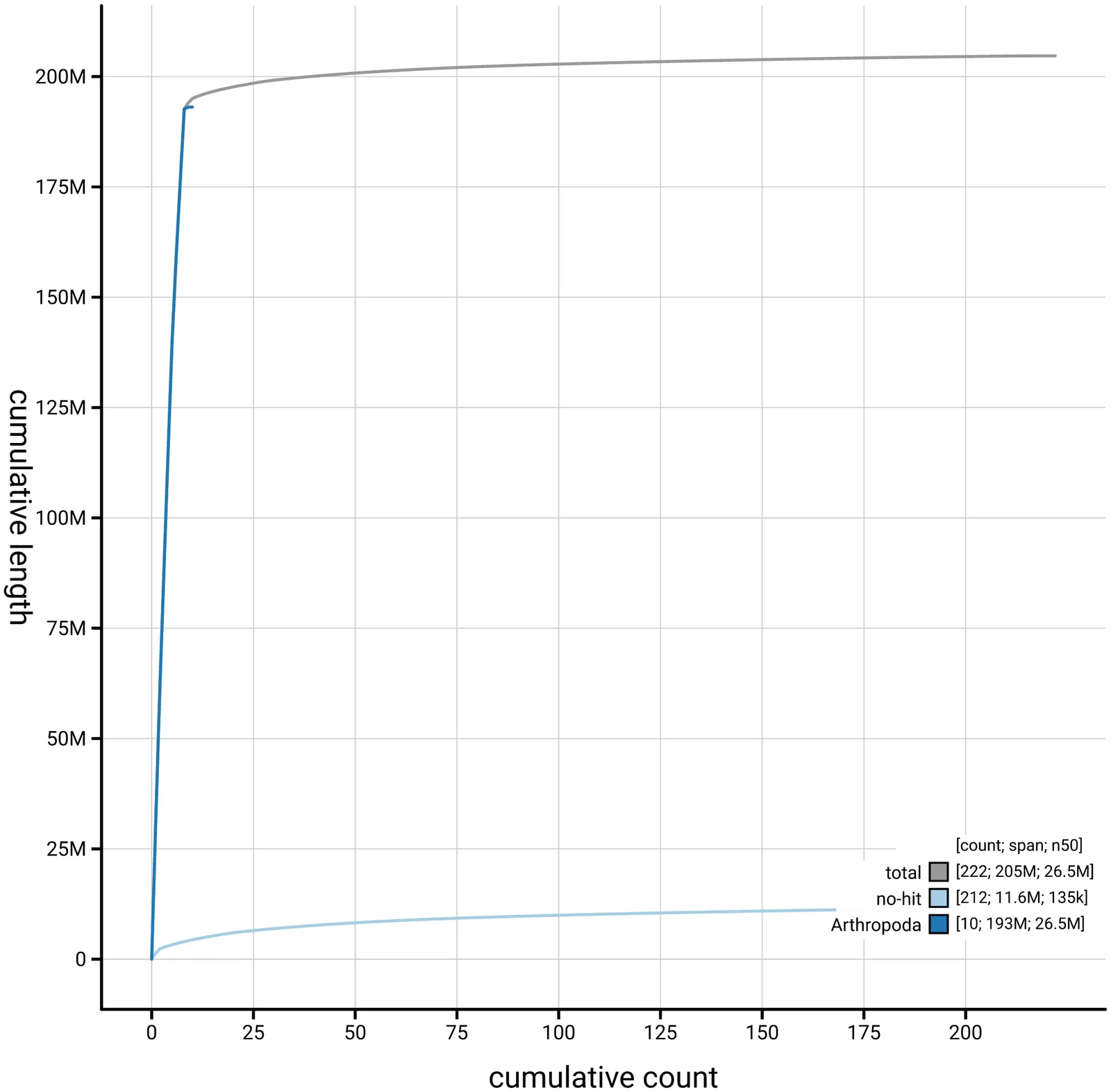
Genome assembly of *Arge pagana* iyArgPaga1.1: BlobToolKit cumulative sequence plot. The grey line shows cumulative length for all scaffolds. Coloured lines show cumulative lengths of scaffolds assigned to each phylum using the buscogenes taxrule. An interactive version of this figure is available at https://blobtoolkit.genomehubs.org/view/GCA_963969455.1/dataset/GCA_963969455.1/cumulative.

The snail plot in Figure 2 provides a summary of the assembly statistics, indicating the distribution of scaffold lengths and other assembly metrics. Figure 3 shows the distribution of scaffolds by GC proportion and coverage, helping to distinguish between various genomic elements. Figure 4 presents a cumulative assembly plot, with separate curves representing different scaffold subsets assigned to various phyla, illustrating the completeness of the assembly.

**Figure 5.**
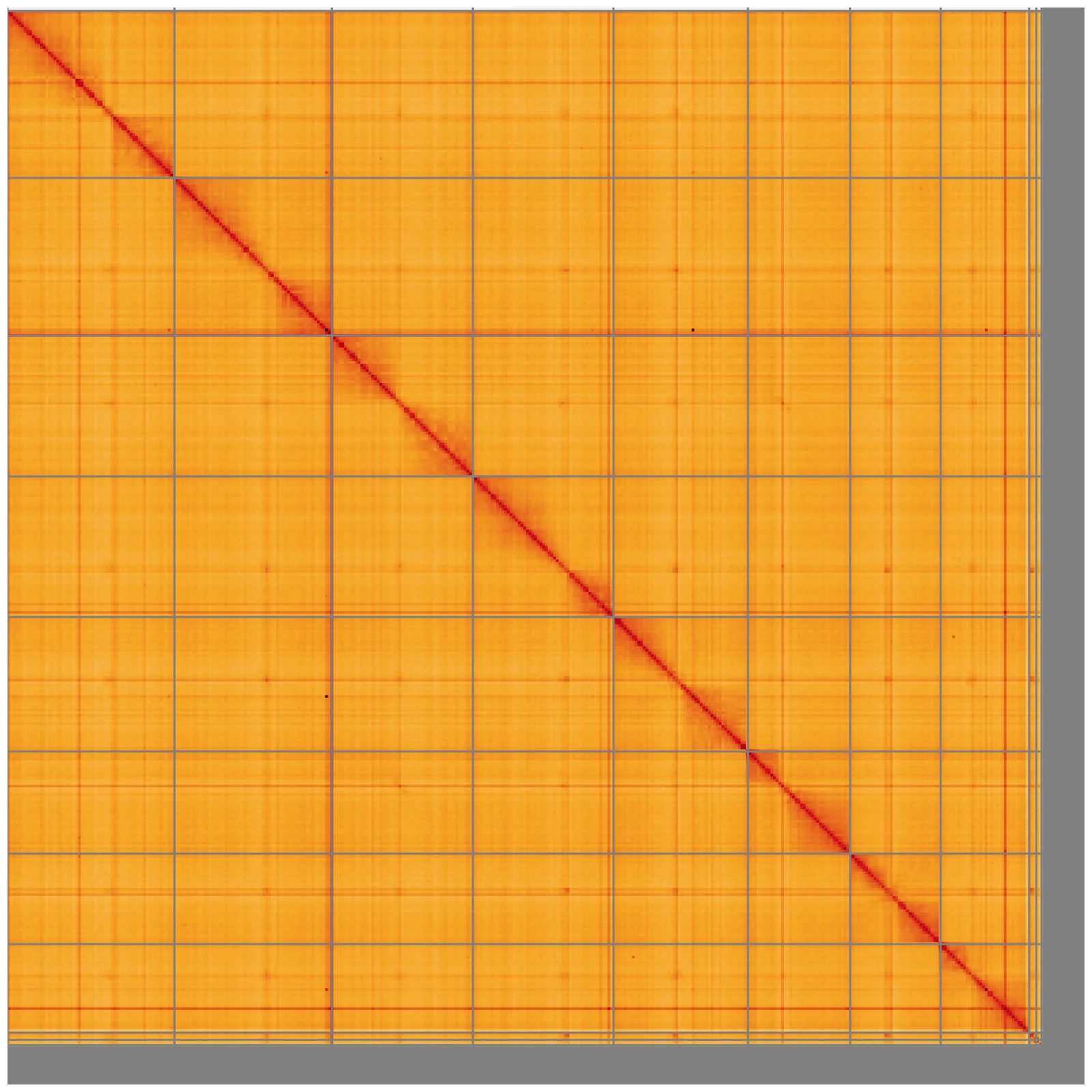
Genome assembly of *Arge pagana* iyArgPaga1.1: Hi-C contact map of the iyArgPaga1.1 assembly, visualised using HiGlass. Chromosomes are shown in order of size from left to right and top to bottom. An interactive version of this figure may be viewed at https://genome-note-higlass.tol.sanger.ac.uk/l/?d=Sp8hozC7RmqlWEgn2YQSpA.

Most (94.4%) of the assembly sequence was assigned to 8 chromosomal-level scaffolds. These chromosome-level scaffolds, confirmed by the Hi-C data, are named in order of size ( Figure 5; Table 3). During manual curation it was noted that the order and orientation of the scaffolds are uncertain in the following regions: Chromosome 1 ~18.80–19.79 Mb, Chromosome 2 ~17.25–17.95 Mb, Chromosome 3 ~12.89–13.42 Mb, Chromosome 5 ~12.11–12.76 Mb, Chromosome 7 ~7.37–8.92 Mb and Chromosome 8 ~ 6.08–6.93 Mb.

**Table 3.** Chromosomal pseudomolecules in the genome assembly of *Arge pagana*, iyArgPaga1.

INSDC accession	Name	Length (Mb)	GC%
OZ018308.1	1	31.5	43.0
OZ018309.1	2	29.69	43.5
OZ018310.1	3	26.58	43.0
OZ018311.1	4	26.51	43.5
OZ018312.1	5	25.36	43.5
OZ018313.1	6	19.27	44.0
OZ018314.1	7	17.07	44.0
OZ018315.1	8	16.68	43.0
OZ018316.1	MT	0.03	19.0

While not fully phased, the assembly deposited is of one haplotype. Contigs corresponding to the second haplotype have also been deposited. The mitochondrial genome was also assembled and can be found as a contig within the multifasta file of the genome submission, and as a separate fasta file with accession OZ018316.1.

The final assembly has a Quality Value (QV) of 55.9 and *k*-mer completeness of 99.99%. BUSCO (v5.4.3) analysis using the hymenoptera_odb10 reference set ( *n* = 5,991) indicated a completeness score of 92.8% (single = 92.5%, duplicated = 0.3%). The assembly achieves the Earth BioGenome Project reference standard of 5.7.Q56. Other quality metrics are given in Table 2. 

Metadata for specimens, BOLD barcode results, spectra estimates, sequencing runs, contaminants and pre-curation assembly statistics are given at https://links.tol.sanger.ac.uk/species/1384777.

## Methods

### Sample acquisition and DNA barcoding

An adult female specimen of *Arge pagana* (specimen ID Ox001705, ToLID iyArgPaga1) was collected from Wytham Woods, Berkshire, United Kingdom (latitude 51.77, longitude –1.34) on 2021-07-22 by netting. The specimen was collected and identified by Liam Crowley (University of Oxford) and preserved on dry ice.

The initial identification was verified by an additional DNA barcoding process according to the framework developed by Twyford *et al*. (2024). A small sample was dissected from the specimen and stored in ethanol, while the remaining parts were shipped on dry ice to the Wellcome Sanger Institute (WSI). The tissue was lysed, the COI marker region was amplified by PCR, and amplicons were sequenced and compared to the BOLD database, confirming the species identification ( Crowley *et al.*, 2023). Following whole genome sequence generation, the relevant DNA barcode region was also used alongside the initial barcoding data for sample tracking at the WSI ( Twyford *et al.*, 2024). The standard operating procedures for Darwin Tree of Life barcoding have been deposited on protocols.io ( Beasley *et al.*, 2023).

### Nucleic acid extraction

The workflow for high molecular weight (HMW) DNA extraction at the Wellcome Sanger Institute (WSI) Tree of Life Core Laboratory includes a sequence of core procedures: sample preparation and homogenisation, DNA extraction, fragmentation and purification. Detailed protocols are available on protocols.io ( Denton *et al.*, 2023b). The iyArgPaga1 sample was prepared for DNA extraction by weighing and dissecting it on dry ice ( Jay *et al.*, 2023), and tissue from the abdomen was homogenised using a PowerMasher II tissue disruptor ( Denton *et al.*, 2023a).

HMW DNA was extracted in the WSI Scientific Operations core using the Automated MagAttract v2 protocol ( Oatley *et al.*, 2023). The DNA was sheared into an average fragment size of 12–20 kb in a Megaruptor 3 system ( Bates *et al.*, 2023). Sheared DNA was purified by solid-phase reversible immobilisation, using AMPure PB beads to eliminate shorter fragments and concentrate the DNA ( Strickland *et al.*, 2023). The concentration of the sheared and purified DNA was assessed using a Nanodrop spectrophotometer and Qubit Fluorometer using the Qubit dsDNA High Sensitivity Assay kit. Fragment size distribution was evaluated by running the sample on the FemtoPulse system.

### Hi-C preparation

Tissue from the head and thorax of the iyArgPaga1 sample was processed at the WSI Scientific Operations core, using the Arima-HiC v2 kit. In brief, frozen tissue (stored at –80°C) was fixed, and the DNA crosslinked using a TC buffer with 22% formaldehyde. After crosslinking, the tissue was homogenised using the Diagnocine Power Masher-II and BioMasher-II tubes and pestles. Following the kit manufacturer's instructions, crosslinked DNA was digested using a restriction enzyme master mix. The 5’-overhangs were then filled in and labelled with biotinylated nucleotides and proximally ligated. An overnight incubation was carried out for enzymes to digest remaining proteins and for crosslinks to reverse. A clean up was performed with SPRIselect beads prior to library preparation.

### Library preparation and sequencing

Library preparation and sequencing were performed at the WSI Scientific Operations core. Pacific Biosciences HiFi circular consensus DNA sequencing libraries were prepared using the PacBio Express Template Preparation Kit v2.0 (Pacific Biosciences, California, USA) as per the manufacturer's instructions. The kit includes the reagents required for removal of single-strand overhangs, DNA damage repair, end repair/A-tailing, adapter ligation, and nuclease treatment. Library preparation also included a library purification step using AMPure PB beads (Pacific Biosciences, California, USA) and size selection step to remove templates shorter than 3 kb using AMPure PB modified SPRI. DNA concentration was quantified using the Qubit Fluorometer v2.0 and Qubit HS Assay Kit and the final library fragment size analysis was carried out using the Agilent Femto Pulse Automated Pulsed Field CE Instrument and gDNA 165kb gDNA and 55kb BAC analysis kit. Samples were sequenced using the Sequel IIe system (Pacific Biosciences, California, USA). The concentration of the library loaded onto the Sequel IIe was between 40–135 pM. The SMRT link software, a PacBio web-based end-to-end workflow manager, was used to set-up and monitor the run, as well as perform primary and secondary analysis of the data upon completion.

For Hi-C library preparation, DNA was fragmented to a size of 400 to 600 bp using a Covaris E220 sonicator. The DNA was then enriched, barcoded, and amplified using the NEBNext Ultra II DNA Library Prep Kit following manufacturers’ instructions. The Hi-C sequencing was performed using paired-end sequencing with a read length of 150 bp on an Illumina NovaSeq 6000 instrument.

### Genome assembly, curation and evaluation

***Assembly***

The HiFi reads were first assembled using Hifiasm ( Cheng *et al.*, 2021) with the --primary option. Haplotypic duplications were identified and removed using purge_dups ( Guan *et al.*, 2020). The Hi-C reads were mapped to the primary contigs using bwa-mem2 ( Vasimuddin *et al.*, 2019). The contigs were further scaffolded using the provided Hi-C data ( Rao *et al.*, 2014) in YaHS ( Zhou *et al.*, 2023) using the --break option. The scaffolded assemblies were evaluated using Gfastats ( Formenti *et al.*, 2022), BUSCO ( Manni *et al.*, 2021) and MERQURY.FK ( Rhie *et al.*, 2020).

The mitochondrial genome was assembled using MitoHiFi ( Uliano-Silva *et al.*, 2023), which runs MitoFinder ( Allio *et al.*, 2020) and uses these annotations to select the final mitochondrial contig and to ensure the general quality of the sequence.

***Assembly curation***

The assembly was decontaminated using the Assembly Screen for Cobionts and Contaminants (ASCC) pipeline (article in preparation). Flat files and maps used in curation were generated in TreeVal ( Pointon *et al.*, 2023). Manual curation was primarily conducted using PretextView ( Harry, 2022), with additional insights provided by JBrowse2 ( Diesh *et al.*, 2023) and HiGlass ( Kerpedjiev *et al.*, 2018). Scaffolds were visually inspected and corrected as described by Howe *et al*. (2021). Any identified contamination, missed joins, and mis-joins were corrected, and duplicate sequences were tagged and removed. The curation process is documented at https://gitlab.com/wtsi-grit/rapid-curation (article in preparation).

***Evaluation of the final assembly***

The final assembly was post-processed and evaluated using the three Nextflow ( Di Tommaso *et al.*, 2017) DSL2 pipelines: sanger-tol/readmapping ( Surana *et al.*, 2023a), sanger-tol/genomenote ( Surana *et al.*, 2023b), and sanger-tol/blobtoolkit ( Muffato *et al.*, 2024). The readmapping pipeline aligns the Hi-C reads using bwa-mem2 ( Vasimuddin *et al.*, 2019) and combines the alignment files with SAMtools ( Danecek *et al.*, 2021). The genomenote pipeline converts the Hi-C alignments into a contact map using BEDTools ( Quinlan & Hall, 2010) and the Cooler tool suite ( Abdennur & Mirny, 2020). The contact map is visualised in HiGlass ( Kerpedjiev *et al.*, 2018). This pipeline also generates assembly statistics using the NCBI datasets report ( Sayers *et al.*, 2024), computes *k*-mer completeness and QV consensus quality values with FastK and MERQURY.FK, and runs BUSCO ( Manni *et al.*, 2021) to assess completeness.

The blobtoolkit pipeline is a Nextflow port of the previous Snakemake Blobtoolkit pipeline ( Challis *et al.*, 2020). It aligns the PacBio reads in SAMtools and minimap2 ( Li, 2018) and generates coverage tracks for regions of fixed size. In parallel, it queries the GoaT database ( Challis *et al.*, 2023) to identify all matching BUSCO lineages to run BUSCO ( Manni *et al.*, 2021). For the three domain-level BUSCO lineages, the pipeline aligns the BUSCO genes to the UniProt Reference Proteomes database ( Bateman *et al.*, 2023) with DIAMOND ( Buchfink *et al.*, 2021) blastp. The genome is also split into chunks according to the density of the BUSCO genes from the closest taxonomic lineage, and each chunk is aligned to the UniProt Reference Proteomes database with DIAMOND blastx. Genome sequences without a hit are chunked with seqtk and aligned to the NT database with blastn ( Altschul *et al.*, 1990). The blobtools suite combines all these outputs into a blobdir for visualisation.

The genome assembly and evaluation pipelines were developed using nf-core tooling ( Ewels *et al.*, 2020) and MultiQC ( Ewels *et al.*, 2016), relying on the Conda package manager, the Bioconda initiative ( Grüning *et al.*, 2018), the Biocontainers infrastructure ( da Veiga Leprevost *et al.*, 2017), as well as the Docker ( Merkel, 2014) and Singularity ( Kurtzer *et al.*, 2017) containerisation solutions.

Table 4 contains a list of relevant software tool versions and sources.

**Table 4.** Software tools: versions and sources.

Software tool	Version	Source
BEDTools	2.30.0	https://github.com/arq5x/bedtools2
BLAST	2.14.0	ftp://ftp.ncbi.nlm.nih.gov/blast/executables/blast+/
BlobToolKit	4.3.7	https://github.com/blobtoolkit/blobtoolkit
BUSCO	5.4.3 and 5.5.0	https://gitlab.com/ezlab/busco
bwa-mem2	2.2.1	https://github.com/bwa-mem2/bwa-mem2
Cooler	0.8.11	https://github.com/open2c/cooler
DIAMOND	2.1.8	https://github.com/bbuchfink/diamond
fasta_windows	0.2.4	https://github.com/tolkit/fasta_windows
FastK	427104ea91c78c3b8b8b49f1a7d6bbeaa869ba1c	https://github.com/thegenemyers/FASTK
Gfastats	1.3.6	https://github.com/vgl-hub/gfastats
GoaT CLI	0.2.5	https://github.com/genomehubs/goat-cli
Hifiasm	0.16.1	https://github.com/chhylp123/hifiasm
HiGlass	44086069ee7d4d3f6f3f0012569789ec138f42b84aa44357826c0b6753eb28de	https://github.com/higlass/higlass
Merqury.FK	d00d98157618f4e8d1a9190026b19b471055b22e	https://github.com/thegenemyers/MERQURY.FK
MitoHiFi	3.2	https://github.com/marcelauliano/MitoHiFi
MultiQC	1.14, 1.17, and 1.18	https://github.com/MultiQC/MultiQC
NCBI Datasets	15.12.0	https://github.com/ncbi/datasets
Nextflow	23.04.0-5857	https://github.com/nextflow-io/nextflow
PretextView	0.2	https://github.com/sanger-tol/PretextView
purge_dups	1.2.3	https://github.com/dfguan/purge_dups
samtools	1.16.1, 1.17, and 1.18	https://github.com/samtools/samtools
sanger-tol/ascc	-	https://github.com/sanger-tol/ascc
sanger-tol/genomenote	1.1.1	https://github.com/sanger-tol/genomenote
sanger-tol/readmapping	1.2.1	https://github.com/sanger-tol/readmapping
Seqtk	1.3	https://github.com/lh3/seqtk
Singularity	3.9.0	https://github.com/sylabs/singularity
TreeVal	1.0.0	https://github.com/sanger-tol/treeval
YaHS	1.2a.2	https://github.com/c-zhou/yahs

### Wellcome Sanger Institute – Legal and Governance

The materials that have contributed to this genome note have been supplied by a Darwin Tree of Life Partner. The submission of materials by a Darwin Tree of Life Partner is subject to the **‘Darwin Tree of Life Project Sampling Code of Practice’**, which can be found in full on the Darwin Tree of Life website here. By agreeing with and signing up to the Sampling Code of Practice, the Darwin Tree of Life Partner agrees they will meet the legal and ethical requirements and standards set out within this document in respect of all samples acquired for, and supplied to, the Darwin Tree of Life Project.

Further, the Wellcome Sanger Institute employs a process whereby due diligence is carried out proportionate to the nature of the materials themselves, and the circumstances under which they have been/are to be collected and provided for use. The purpose of this is to address and mitigate any potential legal and/or ethical implications of receipt and use of the materials as part of the research project, and to ensure that in doing so we align with best practice wherever possible. The overarching areas of consideration are:

•    Ethical review of provenance and sourcing of the material

•    Legality of collection, transfer and use (national and international)

Each transfer of samples is further undertaken according to a Research Collaboration Agreement or Material Transfer Agreement entered into by the Darwin Tree of Life Partner, Genome Research Limited (operating as the Wellcome Sanger Institute), and in some circumstances other Darwin Tree of Life collaborators.

## Data Availability

European Nucleotide Archive: *Arge pagana* (large rose sawfly). Accession number PRJEB66087; https://identifiers.org/ena.embl/PRJEB66087 ( Wellcome Sanger Institute, 2024). The genome sequence is released openly for reuse. The *Arge pagana* genome sequencing initiative is part of the Darwin Tree of Life (DToL) project. All raw sequence data and the assembly have been deposited in INSDC databases. The genome will be annotated using available RNA-Seq data and presented through the Ensembl pipeline at the European Bioinformatics Institute. Raw data and assembly accession identifiers are reported in Table 1 and Table 2.

## Author information

Members of the University of Oxford and Wytham Woods Genome Acquisition Lab are listed here: https://doi.org/10.5281/zenodo.12157525.

Members of the Darwin Tree of Life Barcoding collective are listed here: https://doi.org/10.5281/zenodo.12158331.

Members of the Wellcome Sanger Institute Tree of Life Management, Samples and Laboratory team are listed here: https://doi.org/10.5281/zenodo.12162482.

Members of Wellcome Sanger Institute Scientific Operations: Sequencing Operations are listed here: https://doi.org/10.5281/zenodo.12165051.

Members of the Wellcome Sanger Institute Tree of Life Core Informatics team are listed here: https://doi.org/10.5281/zenodo.12160324.

Members of the Tree of Life Core Informatics collective are listed here: https://doi.org/10.5281/zenodo.12205391.

Members of the Darwin Tree of Life Consortium are listed here: https://doi.org/10.5281/zenodo.4783558.
