# Supplementary material for: The genome sequence of the rose sawfly Arge pagana Linnaeus, 1758
Source: Wellcome Open Res. 2024 Oct 29;9:634. [Version 1] doi: 10.12688/wellcomeopenres.23289.1 (PMC13560640; doi:10.12688/wellcomeopenres.23289.1)
Supplement: Peer review history

# Reviewer report: Reviewer response for version 1

**DOI:** 10.21956/wellcomeopenres.25670.r112424

**Published:** 2024-12-04

**Authors:** Trevor Sless

**Affiliations:** 1 York University, Toronto, Ontario, Canada

The submitted article presents the genome assembly of the sawfly *Arge pagana *for the first time. The methodology includes both PacBio and HiC sequencing, resulting in a high quality chromosome-level assembly, and the methods are explained in detail allowing for replicability. The dataset is also readily accessible, allowing it to serve as a valuable genomic reference for future study. Overall, the paper is a welcome contribution to the field of genomics, particularly with respect to sawflies and other Hymenoptera, and I have no specific corrections.

Are sufficient details of methods and materials provided to allow replication by others?

Yes

Is the rationale for creating the dataset(s) clearly described?

Yes

Are the datasets clearly presented in a useable and accessible format?

Yes

Are the protocols appropriate and is the work technically sound?

Yes

Reviewer Expertise:

Evolution, phylogenetics, genomics, microbiomics

I confirm that I have read this submission and believe that I have an appropriate level of expertise to confirm that it is of an acceptable scientific standard.

# Reviewer report: Reviewer response for version 1

**DOI:** 10.21956/wellcomeopenres.25670.r112422

**Published:** 2024-11-28

**Authors:** Danilo Trabudo do Amaral

**Affiliations:** 1 Universidade Federal do ABC (UFABC), São Paulo, Brazil

This article presents a high-quality genome assembly of *Arge pagana*, a member of the Argidae family, based on a female specimen. The genome has a total length of 204.70 megabases, with 94.4% of the assembly scaffolded into 8 chromosomal pseudomolecules. The mitochondrial genome was also assembled, totaling 26.8 kilobases. The assembly was achieved using advanced sequencing technologies, including PacBio HiFi and Hi-C scaffolding, followed by extensive manual curation to ensure accuracy. The authors also evaluated the assembly's completeness using BUSCO, achieving a score of 92.8%.

 The study's positive aspects include the high-quality genome assembly, the integration of PacBio HiFi reads, Hi-C data, and manual curation (which ensures a robust and accurate assembly), and the detailed description of methods, spanning from DNA extraction to data curation. However, some minor adjustments could improve the manuscript. These include: exploring the biological significance of the findings in more depth; addressing the underutilization of comparative genomics (the study mentions the phylogenetic context but lacks comparisons with related species); clarifying ambiguities in assembly details, such as uncertainties in scaffold orientation for certain regions, and explaining how these gaps might impact downstream analyses.

 Additionally, while the article includes helpful plots, the visual data could be more effectively integrated into the discussion. For instance, the Hi-C contact map (Figure 5) is not referenced in the text, missing an opportunity to connect visual and narrative elements.

 This manuscript represents a significant contribution to hymenopteran genomics. However, addressing these aspects would further enhance its impact.

Are sufficient details of methods and materials provided to allow replication by others?

Yes

Is the rationale for creating the dataset(s) clearly described?

Yes

Are the datasets clearly presented in a useable and accessible format?

Yes

Are the protocols appropriate and is the work technically sound?

Yes

Reviewer Expertise:

genomics and bioinformatics

I confirm that I have read this submission and believe that I have an appropriate level of expertise to confirm that it is of an acceptable scientific standard, however I have significant reservations, as outlined above.
